# Associations between performance on a non‐immersive virtual reality task and structural correlates of Alzheimer’s disease vulnerable brain regions

**DOI:** 10.1002/alz.085996

**Published:** 2025-01-03

**Authors:** Sophia L. Holmqvist, Katie Jobson, Stephanie M. Simone, Molly B. Tassoni, Moira Mckniff, Dennis DeSalme, Takehiko Yamaguchi, Ingrid Olson, Nadine Martin, Tania Giovannetti

**Affiliations:** ^1^ Temple University, Philadelphia, PA USA; ^2^ Suwa University of Sciences, Chino, Nagano Japan

## Abstract

**Background:**

Objective and sensitive measures of everyday function are needed for accurate clinical diagnosis and evaluation of outcomes in clinical trials for dementia. However, most objective everyday function measures are difficult to administer and have not been validated against biomarkers of Alzheimer’s disease (AD) neuropathology. This study evaluated the neuroimaging correlates of a highly sensitive, ecologically valid, and easily implementable performance‐based test of function called the Virtual Kitchen Challenge (VKC). VKC measures have been validated against brain‐derived markers of cerebrovascular burden in past studies, but this study investigated associations between the VKC and AD biomarkers, including cortical thickness/volume of the hippocampus and medial temporal lobe associated areas.

**Methods:**

Twenty (n = 14 healthy control, n = 6 mild cognitive impairment) community‐dwelling and racially diverse older adults aged 60+ completed conventional neuropsychological tests, Functional Activities Questionnaire (FAQ), and VKC, which required completion of simulated breakfast and lunch tasks on a touch‐screen computer. VKC outcomes included completion time and validated measures of performance efficiency (i.e., screen interactions, proportion of time off screen). A global cognitive composite score was calculated as the average t score across neuropsychological tests. The hippocampus, entorhinal cortex, parahippocampal gyrus, inferior temporal cortex, and inferior parietal cortex were examined as Freesurfer‐derived *a priori* regions of interest. Partial correlations were conducted controlling for age and estimated intracranial volume.

**Results:**

VKC outcomes were associated with entorhinal cortical thickness (VKC completion time, *r* = ‐.530, *p* = .024; time spent off screen, *r* = ‐.515, *p* = .029) and inferior temporal lobe thickness (proportion of time spent off screen, *r* = ‐.747, *p*<.001. Conventional measures were associated with bilateral hippocampal volume (global cognitive composite, *r* = .446, *p* = .064; memory composite, *r* = .408, *p* = .092; FAQ, *r* = ‐.538, *p* = .021) and parahippocampal gyrus thickness (global cognitive composite, *r* = .508, *p* = .031; FAQ, *r* = ‐.643, *p* = .004).

**Conclusion:**

In contrast to conventional measures, VKC outcomes were significantly associated with entorhinal and inferior temporal cortex thickness, brain regions that are typically affected early in AD‐disease progression. Overall, the VKC, particularly measures of completion time and proportion of time off screen, holds promise as a highly sensitive and valid, objective measure of everyday function in a range of contexts with racially diverse older adults, including clinical assessment and clinical trials.

